# Regulatory T Lymphocytes in Periodontitis: A Translational View

**DOI:** 10.1155/2018/7806912

**Published:** 2018-04-02

**Authors:** Carla Alvarez, Carolina Rojas, Leticia Rojas, Emilio A. Cafferata, Gustavo Monasterio, Rolando Vernal

**Affiliations:** ^1^Periodontal Biology Laboratory, Faculty of Dentistry, Universidad de Chile, Santiago, Chile; ^2^Oral Pathology Laboratory, Faculty of Dentistry, Universidad Andrés Bello, Santiago, Chile; ^3^Translational Allergy and Immunology Laboratory, Faculty of Medicine, Pontificia Universidad Católica de Chile, Santiago, Chile; ^4^Dentistry Unit, Faculty of Health Sciences, Universidad Autónoma de Chile, Santiago, Chile

## Abstract

Periodontitis is a chronic immuno-inflammatory disease in which the disruption of the balance between host and microbiota interactions is key to the onset and progression of the disease. The immune homeostasis associated with periodontal health requires a regulated immuno-inflammatory response, during which the presence of regulatory T cells (Tregs) is essential to ensure a controlled response that minimizes collateral tissue damage. Since Tregs modulate both innate and adaptive immunity, pathological conditions that may resolve by the acquisition of immuno-tolerance, such as periodontitis, may benefit by the use of Treg immunotherapy. In recent years, many strategies have been proposed to take advantage of the immuno-suppressive capabilities of Tregs as immunotherapy, including the *ex vivo* and *in vivo* manipulation of the Treg compartment. Ongoing research in both basic and translational studies let us gain a better understanding of the diversity of Treg subsets, their phenotypic plasticity, and suppressive functions, which can be used as a substrate for new immunotherapies. Certainly, as our knowledge of Treg biology increases, we will be capable to develop new therapies designed to enhance the stability and function of Tregs during periodontitis.

## 1. Introduction

Periodontitis is a chronic immuno-inflammatory disease in which the disruption of the balance between host and microbiota interactions plays a pivotal role in the onset and progression of the disease. The host immune response is disturbed by key pathogens, such as *Porphyromonas gingivalis* and *Aggregatibacter actinomycetemcomitans*, and further sustained by pathogenic microorganisms [1]. During periodontitis, the immuno-inflammatory infiltrate invades deep compartments of the periodontium, causing the destruction of the tooth supporting tissues: radicular cement, periodontal ligament, and alveolar bone. In severe cases of the disease, the tissue destruction leads to tooth mobility and ultimately tooth loss [2]. The immune homeostasis associated with periodontal health requires a regulated immuno-inflammatory response, during which the presence of immune regulatory cells is essential to ensure a response that minimizes collateral tissue damage [3]. Tregs are a subset of CD4^+^ T lymphocytes that play an essential role in the maintenance of self-tolerance and immune homeostasis [4]. Although different T cell types with regulatory functions have been identified, the most physiologically relevant Treg population is characterized as CD4^+^ T lymphocytes that constitutively express the IL-2 receptor *α*-chain, CD25, and the transcription factor Foxp3, comprising approximately 10% of the CD4^+^ T cell compartment [5]. Since Tregs modulate both innate and adaptive immunity, pathological conditions that may resolve by the acquisition of immuno-tolerance, such as autoimmune diseases, organ transplantation, and overall inflammatory diseases—including periodontitis—may benefit by the use of Treg immunotherapy [6]. In this context, increasing evidence about *ex vivo* strategies to isolate, preserve, expand, and transfer Tregs, and new protocols to manipulate the Treg pool *in vivo*, have led the way to new promising therapeutic approaches that may be eventually suitable for clinical use.

## 2. Treg Biology

### 2.1. Development

The two main subsets of Tregs are classified accordingly to their site of development. Firstly, natural or thymic Tregs (nTregs or tTregs) develop in the thymus through intermediate strength interactions between a self-reacting T cell receptor (TCR) and their cognate antigens, presented by medullary thymic epithelial cells and hematopoietic antigen-presenting cells, leading to upregulation of CD25 [7, 8]. In addition, costimulatory molecules such as CD28, GITR, OX40, and TNFR2 contribute to Treg development [9]. Most intercellular signals converge to the NF-*κ*B pathway, which appears to be the main transcription factor involved in thymic generation of Tregs [10]. At the terminal stage of differentiation, the transcription factor Foxp3 is upregulated by the action of IL-2 through CD25, whose signalization induces further CD25 production and high expression of suppressor genes, rendering regulatory functions [7]. Compared with induced Tregs, nTregs exhibit a higher expression of PD-1 (programmed cell death-1), neuropilin 1 (Nrp1), Helios, and CD73 [11].

The second route for Treg generation is the differentiation from naïve CD4^+^ T lymphocytes at the periphery, named induced or peripheral Tregs (iTregs or pTregs). iTregs are mostly present in the mucosal interface, by the action of tolerogenic antigen-presenting cells [12]. TGF-*β* is a major inductor of Foxp3 expression through phosphorylation and activation of the transcription factors Smad2 and Smad3, which bind the intronic enhancer CNS1 in the *foxp3* gene locus. In the gut, TGF-*β* is produced by CD103^+^ mucosal dendritic cells (DCs) that also produce retinoic acid (RA), which in turn induces the binding of RA receptor (RAR) and retinoic X receptor (RXR) to CNS1, leading to increased binding of Smad3 [12, 13]. Moreover, gut commensal bacteria also promote the iTreg generation by metabolites, such as short-chain fatty acid (SCFA), secreted from bacterial fermentation of dietary fibers [13–16].

### 2.2. Phenotypic Characterization

The transcription factor Foxp3 is considered the main Treg phenotype marker. Foxp3 stabilized the Treg canonical genetic profile, controlling its differentiation, maintenance, and suppressive functions [17, 18]. However, Foxp3 expression is not exclusive of Tregs, especially in humans, since activated conventional T cells may transiently upregulate Foxp3 without the acquisition of suppressive functions [19]. Besides, Foxp3 is a nuclear protein that cannot be used as a marker to purify viable Tregs; thus, a number of surface phenotypic markers have been characterized to define Tregs and its subsets [20]. In humans, the markers CD25^high^ and CD127^low/−^ are frequently used for Treg sorting from peripheral blood and tissues [21]. Postsorting analysis of this population shows a Foxp3 expression above 87%, indicating a reliable strategy to purify viable Tregs [22]. In addition, several studies have identified phenotypic markers within the CD25^high^ CD127^low^ Foxp3^+^ population that are differentially expressed by discrete Treg subsets, according to activation and memory status (CD45RA naïve and CD45RO memory), chemotactic profile (chemokine receptors like CCR4 and CCR9), suppressive functions (CTLA-4, CD39, and CD73), and more [20]. Definitely, human Tregs are phenotypically complex, and, as technology advances, even more new Treg subtypes have been identified, reaching up to 22 distinct subpopulations [22].

### 2.3. Phenotypic Plasticity

Recent studies suggest that Tregs retain lineage plasticity, the ability to switch their cell fate to other effector T cell subset under particular environmental conditions, such as sustained inflammation or lymphopenia [23]. iTregs have been shown to be more unstable than nTregs. Epigenetic changes in the CNS2 region of the *foxp3* locus explain at least in part this difference. In nTregs, CpG islands of the Treg-specific demethylated region (TSDR) from the CNS2 region are hypo-methylated, but in freshly generated iTregs, this region is heavily methylated; thus, important transcription factors cannot be recruited to the site, and Foxp3 expression becomes unstable [13]. Also, homeostatic proliferation in the periphery depends on cytokines, particularly IL-2 [7]. Pathogenic conversion of Foxp3^+^ T cell into Th17 cells has been demonstrated under inflammatory conditions enriched in IL-6 *in vivo*, where CD25^low^ Foxp3^+^ CD4^+^ T cells lose Foxp3 expression and trans-differentiate into Th17 lymphocytes [24]. Moreover, under certain circumstances, Foxp3^+^ T cells may acquire effector T cell-like features without losing Foxp3 expression, with “hybrid” phenotypes. For example, Foxp3^+^ RORC2^+^ IL-17^+^ cells have been identified in human intestine and Foxp3^+^ T-bet^+^ IFN-*γ*^+^ cells in patients with chronic inflammatory diseases, although in most cases those Foxp3^+^ T cells retain suppressive functions [23].

## 3. Treg Suppressive Mechanisms

The main function of Tregs is the suppression of naïve T cell activation and expansion; however, they can also inhibit activated effector T cells, memory CD4^+^ T cells, CD8^+^ T cells, NKs, NKTs, APCs, and B cells [7].

Tregs present a battery of suppressive mechanisms that may proceed by four distinctive ways: (1) modulation of antigen-presenting cell (APC) maturation or function, (2) suppression by killing targeted cells, (3) suppression by metabolic disruption, and (4) suppression by inhibitory cytokines [25]. An example of APC inhibition is the Treg expression of CTLA-4, an inhibitory receptor relative to the T cell costimulatory molecule CD28. While CD28 signaling promotes T cell activation, CTLA-4 suppresses the T cell response by interacting with costimulatory receptors CD80 and CD86, expressed at the APC surface. This contact leads to the downregulation and sequestration of both costimulatory molecules [26]. Additionally, CTLA-4 induces the expression of the enzyme indoleamine 2,3-dioxygenase (IDO) by DCs, which catalyzes degradation of the essential amino acid tryptophan to kynurenine, leading to effector T cell starvation [25]. Tregs may also kill their target cells through cell contact-dependent cytolysis by granzymes A and B, in both perforin-dependent and perforin-independent manner, or induce apoptosis via the tumor necrosis factor-related apoptosis-inducing ligand-death receptor 5 (TRAIL-DR5) pathway, among other means [25]. Tregs mediate suppressive metabolic disruption of effector T cells by consumption of local IL-2, which limits T cell proliferation [27]. Another suppressive mechanism is the expression of surface ectoenzymes, CD39 and CD73, which catalyze extracellular ATP hydrolysis to ADP, AMP, and adenosine. Adenosine signals may inhibit APCs as well as activated T lymphocytes by elevation of intracellular cAMP [7, 28]. A contact-independent suppressive mechanism is the production of inhibitory cytokines, such as IL-10, IL-35, and TGF-*β*, which interact with their specific receptors in a wide range of cell phenotypes [7].

Another immuno-suppressive function of Tregs is the inhibition of osteoclast differentiation and their bone-resorptive activity [29]. *In vitro* studies, with human or murine Tregs, have shown that these cells can inhibit the differentiation of monocytes/macrophages to osteoclasts by the secretion of TGF-*β*, IL-4, and IL-10, and by the interaction of CTLA-4 with CD80/86 receptors present in osteoclasts and their precursors [30, 31]. In an *in vivo* study of osteoporosis, it was reported that the adoptive transfer of murine Tregs to Rag1^−/−^ mice, deficient of T cells, increases the total bone mass associated with the decrease in the number of osteoclasts [32]. In addition, in patients with rheumatoid arthritis, nTregs secrete low levels of regulatory cytokines and have defects in the expression of CTLA-4, which is associated with increased bone destruction [29]. Therefore, Treg may present important suppressive functions during inflammation-mediated bone destruction, as well as in bone homeostasis.

**Figure 1:**
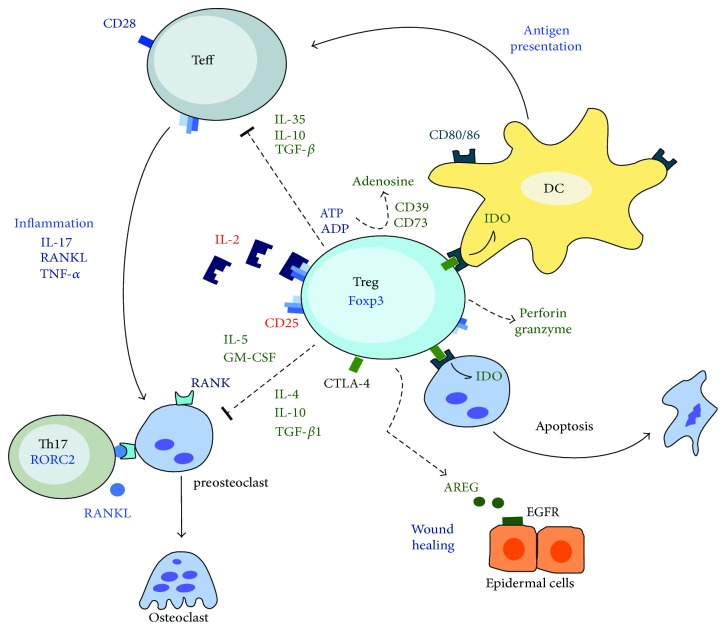
Treg suppressive functions. Tregs have several suppressive mechanisms that may inhibit different cell types. For instance, Tregs can directly inhibit antigen-presenting cells (APCs), such as dendritic cells (DCs), through its inhibitory receptor CTLA-4 that bounds to the costimulatory molecules CD80 and CD86, expressed on the surface of APCs. This interaction induces the production of indoleamine 2,3-dioxygenase (IDO), which in turn may provoke apoptosis of DCs and preosteoclasts. In addition, CTLA-4 competes with the CD28 receptor present on the surface of effector T cells (Teff) and inhibits costimulatory signals during antigenic presentation. Similarly, CTLA-4 directly suppresses osteoclast differentiation and activation, mechanisms potentiated by the secretion of inhibitory cytokines such as IL-4, IL-5, IL-10, TGF-*β*, and GM-CSF. Furthermore, Tregs suppress the pro-inflammatory functions of Teff, such as Th17 (CD4^+^ RORC2^+^) lymphocytes through various mechanisms such as the local consumption of IL-2; secretion of anti-inflammatory cytokines such as IL-10, IL-35, and TGF-*β*; inhibition of antigenic presentation; transformation of ATP and ADP to adenosine by surface ectoenzymes (CD39 and CD73), and the controlled release of perforin and granzyme. Finally, Tregs may promote tissue repair through the production of amphiregulin (AREG), ligand of the epidermal growth factor receptor (EGFR), expressed in epidermal cells and other resident cells.

Besides the immuno-suppression activity, it has been postulated that Tregs may have the capacity to directly exert tissue-repairing functions by promoting the wound healing processes at multiple tissue sites [33]. Tregs, exposed to inflammatory conditions during skin injury, express the epidermal growth factor receptor (EGFR), which plays a major role in skin wound healing by stimulating epidermal and dermal regeneration. Specific ablation of Tregs early after the skin injury resulted in delayed wound reepithelialization and closure, increased granulation tissue, and bigger overlying eschar [34]. Also, in a murine model of infectious lung injury, Tregs produce amphiregulin, an EGFR ligand, in response to the inflammatory cytokines IL-18 and IL-33. This Treg effect is independent of TCR signaling and dispensable for their suppressive functions, indicating a distinct tissue-protective function that is evoked in response to specific cues, different from those that induce suppressive functions [35] (Figure 1).

## 4. Tregs in Periodontitis

Although the microorganisms that comprise the pathogenic subgingival biofilm are the primary etiological agents of periodontitis, the determinant of the disease progression and clinical outcome is the host's immune response, which includes the formation of the periodontal inflammatory infiltrate and the activation of osteoclasts [36]. During periodontitis, the immune response has to be controlled to effectively avoid the pathogenic microorganism dissemination and, at the same time, prevent collateral tissue damage. Therefore, Tregs preferentially accumulate at infected tissues, limiting the immune responses and promoting pathogen survival [37]. Different studies have described the enrichment of Tregs within the infected periodontal tissues. For instance, there is a higher frequency of CD4^+^ CD25^+^ CTLA-4^+^ Tregs in periodontitis biopsies than in gingivitis [36]. These cells exhibited phenotypic characteristics of Tregs, confirmed by the expression of CTLA-4, GITR, CD103, CD45RO, and Foxp3 [37]. Moreover, the migration of CD4^+^ CD25^+^ T cells to periodontitis that affected gingival tissues seemed to be dependent on CCL17 and CCL22 expression by the local inflammatory infiltrate, which recruits Treg expressing CCR4 or CCR8 [37, 38]. Despite the increase in the number of Tregs during periodontitis, it is possible that a fraction of these cells loses their suppressive functions due to the inflammatory periodontal environment enriched in IL-6 [39]. For instance, in active periodontal lesions, compared with inactive lesions, Foxp3, T-bet, RANKL, IL-17, IL-1*β*, and IFN-*γ* mRNAs were significantly overexpressed [40]. However, TGF-*β*1 and IL-10 mRNA expression was increased within inactive periodontal lesions compared to active ones [40]. CD25^+^ Foxp3^+^ Tregs are strikingly diminished in bone resorption lesions from periodontitis compared to healthy gingival tissues. Also, in periodontal tissue homogenates, the correlation between RANKL and IL-10 protein concentrations is negative, whereas the correlation between RANKL and the pro-inflammatory cytokine IL-1*β* is positive [41]. Furthermore, a population of Foxp3^+^ IL-17^+^ cells has been identified in periodontal lesions of patients with periodontitis, indicating the possible conversion of Tregs to Th17 lymphocytes [42]. However, until now, it has not been confirmed whether the periodontal inflammatory environment modifies to some degree the phenotypic or functional stability of infiltrating Tregs.

Different studies using animal models of periodontitis have ratified the importance of Treg suppressive functions during the late stages of the disease. For instance, the inhibition of Tregs with anti-GITR in an *A. actinomycetemcomitans*-induced model of periodontitis showed increased alveolar bone loss associated with the reduction of IL-10, CTLA-4, and TGF-*β* levels [43]. A similar effect was observed in an IDO knockout mouse model in conjunction with lipopolysaccharide- (LPS-) induced gingival inflammation [44]. In this study, the deficiency of IDO increased the number of IL-17^+^ cells and apoptotic or necrotic gingival cells. Also, the number of Tregs was markedly reduced [44]. In a different murine study, Tregs seem to cooperate with Th2 cells, where the coexistence and expression of IL-4, Foxp3, and IL-10 correlate with attenuation of osteolysis. For instance, IL-4 induces CCL22 expression that modulates CCR4-dependent Treg migration. Specifically, experimental periodontitis in IL-4 knockout mice shows an almost total reduction of Tregs and CCL22 production/expression [38]. Therefore, Treg functionality is needed to sustain a controlled immune response that might avoid the disease progression or reactivation.

## 5. Tregs as Therapeutic Tool

In recent years, many strategies have been proposed to take advantage of the immuno-suppressive capabilities of Tregs as immunotherapy [6]. Some of these approaches include the *ex vivo* manipulation of Tregs for adoptive-transfer purposes. In this scheme, Tregs are purified from the host's or a donor's peripheral or banked umbilical cord blood [45]. Subsequently, Tregs are expanded *in vitro* following particular protocols that may include the following: the cell expansion in presence of anti-CD3/CD28 microbeads and rhIL-2, which results in Tregs with polyclonal reactivity [45]; the cell expansion in presence of donor APCs, which generate alloantigen-specific Tregs [46]; or the cell expansion in presence of genetically modified K562-based artificial APCs, which may efficiently expand a specific Treg population [6]. Finally, the developed Tregs may be phenotyped and infused in the patient. So far, numerous clinical trials, most of them in pilot safety and feasibility phase, are analyzing the therapeutic effects of Treg infusion in patients with liver transplantation, graft versus host disease, type 1 diabetes mellitus (T1DM), lupus, and auto-immune hepatitis, showing promising results [21, 47, 48].

Another form of immunotherapy is the manipulation of the Treg compartment *in vivo*, by the use of an array of systemically or locally delivered molecules that promote Treg proliferation, phenotype stability, and functionality [6]. Some of the molecules that might affect the Treg pool *in vivo* or *in vitro* are as follows.

### 5.1. Cytokines

Multiple cytokines have been associated with Treg phenotypic stability and suppressive functionality enhancement through different mechanisms. In mice and humans, IL-2 maintains Treg function by stabilizing Foxp3 expression and regulating key Treg-signature molecules such as CTLA-4 and GITR [49]. IL-2 signaling is also essential to prevent the polarization of Tregs into pro-inflammatory effector cells [50]. Besides its direct effect on Foxp3 expression, IL-2 acts indirectly as it is required for negative regulation of the TBX21 and RORC2 loci, which encode two transcription factors that feedback to diminish Foxp3 expression [50]. The potential role of exogenous IL-2 for Treg survival and Foxp3 expression maintenance has led the exploration of therapeutic approaches. Preclinical studies have shown that the delivery of IL-2/anti-IL-2-antibody complexes stimulates Treg expansion and reduces disease in models of T1DM, experimental autoimmune encephalomyelitis (EAE), collagen-induced arthritis, and angiotensin II-induced aortic stiffening [49–51]. In clinical trials, therapy with low-dose IL-2 for the treatment of graft versus host disease (GVHD) and T1DM appears to successfully expand the circulating Treg cell pool [47, 52, 53].

On the other hand, IL-33, a member of the IL-1 cytokine family, has recently gained interest as regulator of Treg biology. It binds to ST2 receptor, whose deficiency is associated with augmented inflammatory response [54]. IL-33 has shown to increase the CD4^+^ Foxp3^+^ Treg pool, enhance their suppressive activity, and boost ST2 surface expression [55]. It supports direct and indirect Treg cell expansion through the induction of myeloid cells to secrete IL-2, which increases ST2 expression by T lymphocytes [56]. Moreover, IL-33 has the ability to induce regulatory phenotype by promoting the expansion of ST2^+^ Tregs [57, 58]. The upregulation of ST2 expression on Tregs increases the expression of Foxp3, and it has been suggested that genes encoding both of these molecules might depend on each other [59]. In animal models of skin transplant [60], collagen-induced arthritis [61] treated with IL-33 showed the induction of Treg proliferation and enhancement of their immuno-suppressive properties. Furthermore, in patients with type 1 diabetes, *in vitro* IL-33 treatment induced regulatory CD4^+^ CD25^high^ FOXP3^+^ cell frequencies as well as upregulated the surface expression of ST2 molecule and Foxp3 expression [55].

### 5.2. All-*trans* Retinoic Acid

All-*trans* retinoic acid (atRA) is the main active metabolite of vitamin A, well known for playing a major role in various cellular functions, such as proliferation, embryogenesis, differentiation, inflammation, and cell death [62]. Recent studies have revealed that atRA, after binding to RAR, regulates reciprocal differentiation between Tregs and Th17 lymphocytes, reinforcing the regulatory functions of Tregs and suppressing the pro-inflammatory activities of Th17 cells [63, 64]. In conjunction with TGF-*β*1 and IL-2, atRA has shown to improve the differentiation of naïve T lymphocytes into Tregs, reflecting an increment in the number of these cells as well as amplified expression of Foxp3 [65, 66]. atRA enhances the differentiation and stability of iTregs, increasing the activation of the ERK1/2 signaling pathway, resulting in a more stable Foxp3 expression [67]. Moreover, it can increase the nTreg stability under inflammatory conditions through the inhibition of the methylation of the *foxp3* gene [67, 68]. In addition, atRA greatly reduces ROR*γ*t expression and Th17 cell differentiation [69].

### 5.3. Rapamycin

Rapamycin (RAPA) is a macrolide immunosuppressant, widely used in the treatment of organ rejection after transplantation, cancer, and autoimmune diseases [70]. RAPA binds to the mammalian target of rapamycin (mTOR) and inhibits its signaling pathway. The inhibition of mTORC1 and mTORC2 after prolonged exposure to RAPA allows preferential expansion and function of CD4^+^ CD25^hi^ Foxp3^+^ Tregs [71] and blocks critical effector T cell functions such as proliferation, migration, and cytokine production, limiting their differentiation [72]. Conversely, Foxp3 expression by Tregs induces the serine/threonine kinase Pim-2 pathway, which permits the evasion of many RAPA-imposed signaling block [73].

It has been shown that both RAPA and atRA have similar effects on promoting and stabilizing Tregs during their expansion [74]. Although atRA, compared with RAPA, has demonstrated to be more efficient in stabilizing nTregs under inflammatory conditions [67, 75], atRA in conjunction with RAPA promotes the expansion of functional Tregs in the absence of exogenous TGF-*β* [76]. In fact, it has been suggested that the combined use of RAPA and atRA in Treg culture increases the percentage of Tregs with demethylated *foxp3* alleles, making them more likely to remain as Tregs once reinfused in the patient and provide long-lasting, effective control [76]. Adoptive transfer of Tregs after *ex vivo* treatments with atRA and/or RAPA has been considered a promising strategy for cell-based therapeutic treatment of transplant rejection and autoimmune diseases, such as T1DM [21], rheumatoid arthritis [77], and Crohn's disease [78].

### 5.4. Vitamin D

Vitamin D metabolites have long been recognized as important immuno-modulators that exert their functions by binding to the vitamin D receptor (VDR), expressed on many immune cells [79]. The active form of vitamin D, 1*α*,25-dihydroxyvitamin D3 (1,25-[OH]_2_D3, calcitriol), is a secosteroid hormone that is mainly produced by a sunlight-catalyzed biosynthesis pathway in the skin [80]. VDR is a member of the superfamily of hormone nuclear receptors that, after binding with calcitriol, has a conformational change that results in binding to RXR, forming a heterodimer that translocates to the nucleus, where it binds to vitamin D response elements [80]. Calcitriol promotes the growth of Tregs, inhibits Th17 lymphocytes, and induces the secretion of anti-inflammatory cytokines [81]. Moreover, vitamin D also inhibits effector T cell responses through modulation of APC functionality. Treatment of human DCs with calcitriol *in vitro* results in an immature phenotype, known as tolerogenic DCs, characterized by reduced expression of CD80, CD86, and HLA-DR [80, 82]. In this context, pretreatment of human blood-derived myeloid DCs with calcitriol, and then coculture with T cells, inhibits the effector T cell cytokine production and promotes Treg suppressive functions [82].

### 5.5. Controlled Delivery of Treg Promoters

**Figure 2:**
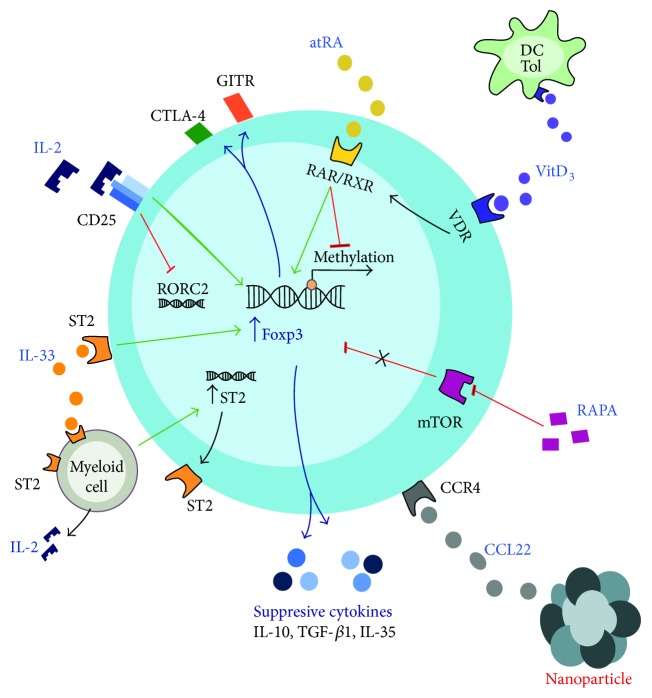
Therapeutic approaches for Treg enrichment *in vivo*. There is an array of systemically or locally delivered molecules that may promote Treg proliferation, phenotype stability, and functionality *in vivo*. Among them, IL-2 has been described as a major growth factor for T cells, particularly important for Treg physiology. IL-2 binds to its receptor CD25, whose signaling pathway induces Foxp3 expression on Tregs and inhibits Th17 differentiation. Foxp3 enables the expression of canonical Treg features, such as CTLA-4, GITR, and anti-inflammatory cytokines. On the other hand, all-*trans* retinoic acid (atRA) and calcitriol (VitD_3_), active metabolites of vitamins A and D, have shown to reinforce suppressive functions of Tregs. atRA induces the binding of the RA receptor (RAR) and the retinoic X receptor (RXR) to an intronic enhancer of *foxp3* gene locus, increasing its expression. Similarly, VitD_3_ binds to the vitamin D receptor (VDR), which later binds to RXR, forming a heterodimer that translocates towards the nucleus to promote Foxp3 expression. Also, VitD_3_ induces tolerogenic dendritic cells (DC Tol), with an immature phenotype that may drive Treg responses. Rapamycin (RAPA) inhibits mTOR signaling pathway, allowing preferential expansion of Tregs, and blocks critical Teff functions. Additionally, IL-33 binds to ST2 receptor, promoting further Foxp3 and ST2 expression on Tregs. Also, indirectly, IL-33 supports Treg expansion, inducing IL-2 secretion by myeloid cells, which stimulates additional ST2 expression. Finally, CCL22-loaded nanoparticles may recruit CCR4^+^ Tregs locally, decreasing Teffs and their pro-inflammatory functions.

The use of the polymeric-nanoparticle technology has demonstrated its biomedical potential due to its ability to encapsulate and control the release of hydrophobic, small molecules [83]. In a recent report, biodegradable poly(ethylene glycol)-poly(lactic-*co*-glycolic acid) (PEG-PLGA) microparticles were engineered to release TGF-*β*, RAPA, and IL-2 to locally induce Treg polarization in an *in vivo* model of allergic contact dermatitis [83]. The prophylactic treatment with these microparticles increased the Treg/Teff ratio in the skin draining lymph nodes, suppressing the T cell-mediated delayed-type hypersensitivity and rendering systemic and specific tolerance to contact allergens [84]. In another study, PLGA microspheres encapsulating recombinant mouse CCL22 were formulated to enhance local recruitment of CCR4^+^ Tregs in a murine model of dry eye disease [84]. The results showed that the microsphere treatment successfully prevents the inflammatory symptomatology by increasing the frequency of Tregs and decreasing the Teff in the lacrimal gland [85]. A different approach for Treg generation was the use of antigenic peptides conjugated to poly(lactide-*co*-glycolide) nanoparticles, which provided a platform for tolerance induction in a murine model of multiple sclerosis (relapsing-remitting experimental autoimmune encephalomyelitis). Through this system, tolerogenic antigen-polymer-conjugated nanoparticles can be formulated to incorporate multiple antigens responsible for the pathogenesis of multiple sclerosis and other diseases [86] (Figure 2).

## 6. Treg Therapeutic Potential in Periodontitis

Treg immuno-suppressive mechanisms and tissue-repairing functions are necessary to sustain periodontal health, which make them an interesting potential therapeutic target. Over the recent years, different therapeutic approaches have been attempted in order to increase the number of functional Tregs in periodontal disease. One approach has been the selective chemo-attraction of Tregs to a particular diseased periodontal lesion by the use of CCL22-releasing microparticles [3]. This method successfully reduced bone resorption, as it enhanced the expression of osteogenic, regenerative, and anti-inflammatory markers in the periodontium, and diminished inflammatory cell infiltration in both murine and canine models of periodontitis [87]. Another method employed in a murine model of periodontitis was the oral administration of atRA. This treatment has shown to effectively regulate the Th17/Treg balance by increasing the percentage of CD4^+^ Foxp3^+^ Tregs and reducing the CD4^+^ ROR*γ*t^+^ Th17 lymphocyte frequency [88]. Similarly, the same group studied the effects of oral administration of tamibarotene (Am80), a synthetic RAR agonist with high specificity for RAR*α* and RAR*β*. Retinoid agonists have been shown to inhibit Th17 cell polarization and to enhance Foxp3 expression during the course of inflammatory diseases; besides, they do not present the atRA limitations such as compound's instability, poor bioavailability, and unexpected side effects. In a murine model of periodontitis, Am80 reduced the percentage of CD4^+^ ROR*γ*t^+^ Th17 lymphocytes and increased the percentage of CD4^+^ Foxp3^+^ Tregs in the gingival tissues, cervical lymph nodes, and spleen. Also, Am80 downregulated the mRNA expression of IL-17A, RANKL, MCP-1, IL-6, and IL-1*β* and upregulated the expression of IL-10 and TGF-*β*1 in gingival tissues and cervical lymph nodes [89].

A different approach has been the subcutaneous vaccination with formalin-killed *P. gingivalis*, which protects mice from inflammation and alveolar bone resorption by modulating the Th17/Treg ratio. The vaccinated mice showed significant reduction in the frequencies of total CD4^+^ T and CD4^+^ ROR*γ*t^+^ cells, and a significant increase in the percentage of Tregs from cervical lymph nodes and spleens [90]. Although these studies showed promising results, more research is still needed in order to apply them in humans.

## 7. Concluding Remarks

Treg protective functions during periodontitis have been demonstrated *in vivo.* Ongoing research in both basic and translational studies lets us gain a better understanding of the diversity of Treg subsets, their plasticity, and their function. Certainly, as our knowledge of Treg biology increases, we will be capable developing new therapies designed to enhance the stability and function of Treg during periodontitis.
